# Deferring to Expertise whilst Maintaining Autonomy

**DOI:** 10.1017/epi.2024.3

**Published:** 2025-06-01

**Authors:** Rebecca C. H. Brown

**Affiliations:** Oxford Uehiro Centre for Practical Ethics, University of Oxford, Oxford OX1 1PT, UK

**Keywords:** Expertise, autonomy, medical ethics, epistemic authority, epistemic autonomy, social epistemology

## Abstract

This paper will consider the extent to which patients’ dependence on clinical expertise when making medical decisions threatens patient autonomy. I start by discussing whether or not dependence on experts is *prima facie* troubling for autonomy and suggest that it is not. I then go on to consider doctors’ and other healthcare professionals’ status as ‘medical experts’ of the relevant sort and highlight a number of ways in which their expertise is likely to be deficient. I then consider how this revised picture of medical expertise should lead us to view the potential threat to patient autonomy that results from depending on such ‘experts’. I argue that, whether or not patients are aware of the limitations of medical expertise, in practice it is difficult to do other than defer to medical advice, and this presents a threat to patient autonomy that should be addressed. I conclude by suggesting some ways in which this threat to autonomy might be mitigated.

## 1 Autonomy in medicine

Maintaining and promoting patient autonomy is a central ethical concern within medical practice. There are a number of reasons why clinical encounters can be high stakes with regard to autonomy. This is due to patients being rendered vulnerable – albeit to varying degrees – by a number of factors. First, the patient is typically physically or mentally unwell. Their independence and capacity to live their life as normal (and as they would choose to) may be compromised. They may be anxious regarding their prognosis and the extent to which they can expect to recover, and according to what timeline. As such, when people seek medical care, they may already be in a position of compromised autonomy.

Second, the dynamic between patients and healthcare professionals further renders patients vulnerable. Patients are typically in a position of epistemic deficiency relative to their healthcare provider. Healthcare professionals (doctors, midwives, nurses, and so on) are typically regarded as ‘experts’ (more on which below) in such contexts, with patients in a position of relative ignorance and dependence.^1^ Healthcare professionals are also in a position of professional power since they act as gatekeepers to care and services (such as prescription medicines), and often hold positions of high social status. Such a ‘power imbalance’ places patients in a subordinate, dependent, position relative to healthcare professionals.

Let us not exaggerate this imbalance, or assume it is inevitable: clearly, some patients do not experience threats to their autonomy in the ways described (perhaps they themselves hold privileged social status, are particularly assertive, or know how to ‘work the system’ to ensure their medical preferences are fulfilled). Vulnerability (and the threat it creates for autonomy) comes in degrees and will be experienced to a varying extent by different people. Nonetheless, the twin effects of bodily compromise and subordination to healthcare professionals create vulnerability and explain why autonomy has been such a preoccupation within medical ethics and law.

## 2 A tension between epistemic dependence and autonomy?

Having outlined a few ways in which medical decision-making contexts can present threats to autonomy,^2^ I will now focus on the supposed threat to autonomy that arises from healthcare professionals’ epistemic superiority to patients.

For now, I will assume that, in the context of medical decision-making, healthcare professionals count as ‘experts’ or ‘epistemic authorities’ and patients will count as ‘novices’ or ‘lay-folk’. I will re-visit the reliability of these commonplace assumptions later. For now, expertise can be taken as involving a high degree of competence in a domain at a time (Watson 2020), where this includes having the necessary skills and access to the relevant evidence in order to answer domain-relevant questions reliably or responsibly (Ballantyne 2019). Epistemic authorities are those ‘we conscientiously judge to be our epistemic superiors, that is, people who tend to perform epistemically better than we do in a given domain’. (Jäger (2016) see also Zagzebski (2012)). Zagzebski uses the term ‘conscientious’ in this context to refer to using one’s faculties *to the best of one*’*s ability* to get to the truth (Zagzebski 2012). In contrast, novices (or lay folk – I shall not distinguish between the two here) lack ‘the relevant sufficient evidence and skills to answer the question reliably on their own’. (Ballantyne 2022)

Epistemically speaking, it makes good sense for a novice to defer to an expert on matters where the expert holds the relevant expertise and skills and the novice is lacking. The expert is more likely to hold or form true beliefs, and the novice can improve the accuracy of her beliefs and stand a greater chance of acquiring knowledge if she defers to the expert. Some, such as Zagzebski, argue that lay folk should adopt an epistemic authority’s beliefs (on the relevant matters) immediately upon discovering that the epistemic authority holds those beliefs (the ‘preemption thesis’). The fact that they are an authority gives the subject a reason to adopt their beliefs, to the extent that the authority holds them, which replaces the subject’s pre-existing reasons for holding those beliefs (or conflicting beliefs) (Zagzebski 2012). We do not need to think that epistemic authorities will be right all of the time to think novices should, as a rule, adopt their beliefs. The claim is just that authorities will be right more often than novices and so, over the long run, the strong kind of deference proposed by the preemption thesis is epistemically advised.

This seems to render novices entirely epistemically dependent upon experts, at least within the domains where the experts *are* experts (or are conscientiously judged by the novices to be epistemic authorities). Translating this into the medical context of patients and doctors (and other healthcare professionals), if patients are novices and doctors experts, then patients are entirely epistemically dependent upon doctors. The preemption thesis would even suggest that patients should replace all of their medicine-related beliefs with the doctor’s beliefs, exactly to the extent that the doctor holds them, and only for the reason that the doctor holds them. It is unimportant what the content of the beliefs are because the setup of the expert/novice dynamic means that the patient is presumed to not be able to judge the reliability of any one belief better than the doctor, so long as we remain in the domain within which the doctor is an epistemic authority.

A more moderate form of deference does not require novices to preemptively replace their beliefs (and reasons for beliefs) with the beliefs of the expert (for the reason, alone, that the expert holds them). Jäger (2016) argues we should not ‘unhinge’ our beliefs from good reasons, as preemption suggests. For instance, if we discover that an epistemic authority shares the same beliefs as us, and for the same reasons, we have evidence that our existing reasons are good ones, and that we should attend to them, rather than evidence we should set them aside. By avoiding preemption and instead attending more carefully to the (total) reasons for holding a (graded) belief, we might make our beliefs more likely to be true, and also gain in terms of our overall understanding of the subject (which may be independently epistemically valuable).^3^

Is this, more moderate approach to deference still a worry for autonomy? It still places a heavy emphasis on deferring to experts by adopting their beliefs and allowing our own reasons for adopting different beliefs to carry little weight. If one’s approach to epistemic goods is to think that understanding has a high value, then one will allow a greater degree of deviation from preemption, in the service of promoting understanding at the cost of knowledge. But if one prioritizes knowledge and true beliefs more highly, then the arguments for non-deference become weaker.

Lackey (2018) offers a more distinct alternative to preemption. Describing the approach taken by Zagzebski as an ‘expert-as-authority’ model of expertise, Lackey recommends instead an ‘expert-as-advisor’ model. Rather than judging experts merely according to their track record of forming true beliefs, Lackey suggests we might evaluate them according to broader criteria, such as their ability to communicate clearly and accessibly, enhance our understanding, etc. She rejects preemption for a number of reasons, including the fact it forces novices to adopt authorities’ beliefs even when they are clearly false or outrageous. Instead, Lackey endorses taking expert advisors’ testimony as evidence to be weighed amongst other relevant evidence we possess. Yet even when experts are modelled as advisors rather than authorities, we may still be highly dependent upon them, as Lackey acknowledges:

Sometimes, we may be so out of our depth that deference is nearly guaranteed, while at other times we may be simply looking for a bit of guidance. The crucial point is that from an epistemic point,… we should never entirely screen off our own reasons in a domain when relying on experts, no matter how good they are. (Lackey 2018: 244)

It seems that, in the medical decision-making context, if one wants to prioritize holding true beliefs, then deferring to doctors and other healthcare professionals is about the best we can do. This seems particularly the case in light of empirical evidence that indicates novices are not very good at recognizing the superiority of expert judgement when it differs from their own (for discussion see Ballantyne (2022)). Such evidence suggests that opportunities for integrating our own reasons alongside those of experts (thus gaining understanding *and* knowledge) are rather limited. Whilst patients are not, perhaps, *entirely* epistemically dependent upon doctors, they are *heavily* dependent upon them. Even if patients are not epistemically required to set aside their own reasons for making a particular decision (and replace them with the single reason that the doctor thinks it is a good decision), they may still be required to weigh doctors’ reasons more heavily than their own, even – in fact, *especially* – if they do not understand them.

If autonomy is supposed to involve self-determination, how can patients be autonomous while depending so heavily on doctors’ beliefs and the potentially opaque reasoning, evidence, and skills that result in those beliefs? I will mention three things here that might help to dampen the threat to autonomy of such epistemic dependence. First, autonomy is not distinct from good epistemic practice, but rather, good epistemic practice will be a component of autonomous action. Second, there is general support from scholars of autonomy to avoid exaggerating the extent to which autonomy requires independence. Third, dependence on doctors’ testimony can be understood as dependence on evidence, and consistent with other evidence-based belief forming processes. I’ll say a little more about each of these before re-visiting the question of the extent to which doctors (and other healthcare professionals) should be considered experts, and the implications of this for patient autonomy.

### 2.1 Good epistemic practice is a part of autonomous action

Being autonomous means being self-determining. This involves being able to act in accord with one’s values and not have one’s actions controlled by others. But even if one is not subject to coercion or intentional manipulation by other agents, one who is seriously mistaken about the grounds for her action, perhaps due to holding false beliefs, will lack the capacity to draw connections between her values and her actions. Such an inability to align one’s actions with one’s values would seem to undermine autonomy (Pugh 2020). The extent to which one’s beliefs must be accurate or one’s reasoning processes rational (and what standards of rationality to apply) only emerge upon provision of a fuller account of autonomy. But it is likely that there will be some ‘decisionally necessary’ beliefs that must be true for an agent to count as making an autonomous decision. For instance, a patient must have access to the information that a vasectomy will render him infertile to make an autonomous decision to undergo one, but he needn’t know details about the biomechanics of sperm production (Pugh 2020). He might access this information via expert testimony (see ‘Testimony as Evidence’ below) or via some other source (e.g. the testimony of those who have undergone vasectomies).^4^ Good epistemic practice in the form of evidence seeking and evaluation will tend to promote true beliefs, knowledge, and understanding, and will therefore tend to promote decision-making that aligns with an agent’s values and enables self-determination (and hence autonomy).

### 2.2 Autonomy does not require independence

It is generally accepted that an over-emphasis on independence can mislead us regarding what autonomy requires. Broadly feminist work on autonomy has highlighted how interdependence and caring relationships are perfectly consistent with autonomous lives, and that many would find a life lived free of influence from others to be barren and unfulfilling, in contrast to lives lived entangled in social relationships. As described by Dworkin:

the conception of autonomy that insists upon substantive independence is not one that has a claim to our respect as an ideal… it makes autonomy inconsistent with loyalty, objectivity, commitment, benevolence, and love. (Dworkin 1988)

Dworkin and others propose a procedural approach to understanding autonomy. Rather than insisting upon substantive values such as independence and self-reliance, procedural accounts can remain content-neutral with regard to the preferences and conceptions of the good that may be held by an autonomous individual. Procedural accounts characterize autonomous agents as acquiring their preferences in particular ways: for instance, through critical self-reflection and endorsement of their preferences. There is nothing inconsistent between this understanding of autonomy and one’s preferences emerging from one’s particular social relationships.

In the medical decision-making context, this highlights how family members can facilitate autonomous decisions (as opposed to undermining them). Family can help patients to better understand the implications of different medical decisions for their values in ways that clinical experts cannot, due to family members’ familiarity with the patient and insight into what they are likely to care about. Relatives can also help patients articulate those value-based preferences in the context of clinical encounters.^5^ Such insights counter the initial concern that dependence on another agent’s beliefs, skills, advice, and so on will threaten autonomy.

### 2.3 Testimony as evidence

In discussing epistemic autonomy, Fricker (2021*a*, 2021*b*) considers the extent to which trusting others as sources of knowledge means surrendering control over what one believes (and thus surrendering autonomy). Fricker describes a ‘thin’ account of trust as ‘trust-based reliance’. For Fricker, reliance on a person involves a belief that they will not easily fail to perform a particular action on a particular occasion.^6^ Such reliance emerges from trust when it is based on particular epistemic and/or character virtues. Trust-based reliance on a medical doctor could be justified by their knowledge of the relevant clinical evidence, their conscientiousness in considering possible diagnoses/treatment options, their intellectual humility regarding the extent of their knowledge, their benevolent intentions towards their patient, and so on. Fricker’s account is ‘thin’ insofar as the person being trusted need not be aware that she is being trusted.

Fricker thus treats testimony as we would other kinds of evidence:

there is no reason why one cannot form beliefs through trusting the word of others in accordance with the requirement to form and sustain one’s beliefs in accordance with one’s evidence, and so retain epistemic self-governance. (Fricker 2021*b*: 337)

The reliability of such testimony can be evaluated through meta-evidence (are they trustworthy in the ways we assume? Do they possess the virtues we take them to?), and which we may choose to rely upon or discard according to our own standards of belief formation.^7^ Thus, dependence on doctors’ claims about the health harms or benefits of a particular course of treatment is no different from dependence upon other forms of evidence (say, from clinical trials), and shouldn’t threaten patient autonomy any more than does dependence on these other sources of evidence (which is to say, at all).^8^ Our capacity to evaluate the reliability of others’ testimony, and to exercise control over whose testimony influences our beliefs will be important for autonomy (Wiland 2021).

## 3 Are healthcare professionals experts? Are they epistemic authorities?

Good epistemic practice seems to render patients heavily, if not entirely, dependent on healthcare professionals when making decisions about treatment options. Such dependence seems, at first glance, to threaten autonomy, to the extent that autonomy describes one’s ability to *self*-determine rather than have one’s actions determined by others. Yet a closer look suggests that such dependence need not be a worry for patient autonomy.

The foregoing discussion has assumed that doctors and other healthcare professionals really do count as experts or epistemic authorities in the right way, and I now want to consider the extent to which this assumption is justified.

Recall, epistemic authorities are described by Zagzebski (2012) as those whom the subject conscientiously judges to be authorities for them in a given domain. This comes down to a judgement about the way the epistemic authority has formed her belief (or forms beliefs in this domain in general). We judge people as epistemic authorities when we think that their way of forming beliefs is superior to our own: they are thus more likely than us to form true beliefs (in this domain), and we are more likely to hold true beliefs if we simply adopt theirs, rather than trying to form beliefs based upon our own belief-forming mechanisms.^9^

The notion of ‘expert’ and ‘expertise’ is in more common usage and has received more attention. There are a number of approaches to describing what makes someone an expert in a particular domain at a particular time and I’ll draw here on Goldman’s (2018) and Watson’s (2020) discussions in order to give a brief overview. First, expertise may be a broad social phenomenon. Goldman describes a reputational account whereby experts are those people widely regarded as experts by the communities in which they live. Such approaches are unappealing since they allow any convincing quack to qualify as an expert. Watson summarizes more plausible accounts, such as Turner (2013) and Collins and Evans (2019) which avoid making expertise vulnerable to epistemic relativism, whilst capturing the fact that experts develop and exist in social contexts that shape what it means to be an expert in a domain at a particular time.

Second are what we might call ‘cognitive authority’ approaches, which identify expertise as emerging from and justifying experts’ cognitive authority. This includes truth-linked or veritistic approaches which define experts in a domain as (something like) those who have more true beliefs and fewer false beliefs about propositions in that domain than most people, where the absolute number of relevant true beliefs they have is substantial (Goldman 2018). Watson rejects a veritistic approach and proposes an alternative ‘epistemic facility’ account whereby:

A subject, S, is an expert in a domain, D, if and only if S (a) understands enough of the terms, propositions, arguments, applications, and aims of D, along with the procedures used to formulate meaningful or useful claims or advice in D, such that (b) S has the ability to successfully demonstrate (a) to some relevant population in the discharge of her epistemic activities. (Watson 2020: 236)

Goldman also describes an epistemological approach, where experts are those who possess substantially more/better evidence relevant to a particular domain than most people (Goldman 2018).

Third are approaches described by Goldman as capacity-based. These define experts as those who have the capacity to help others solve problems or execute tasks in a particular domain.

Fourth, and perhaps related to the capacity-based approaches, are what Watson calls performance-based approaches, which focus on the conditions under which expertise is gained, such as through dedicated training in appropriate environments, which allow experts to perform those activities associated with their form of expertise competently.

Watson offers his own general theory of expertise, which combines elements of social role, performance, and cognitive authority, whereby expertise = a high degree of competence in a domain at a time. That competence must be acquired through rigorous training and be confirmed to be high enough by the current state of skills and information currently possessed within the domain (Watson 2020).^10^

‘Expert’ is thus a broader term than epistemic authority. Whilst an epistemic authority describes the relationship between two people – the subject and the (potential) authority – expert describes someone’s social and epistemic standing more generally. So, to what extent should medical professionals be considered epistemic authorities with regard to their patients, or considered as experts more generally?

Without committing to any one approach, we can see how doctors measure up as experts on the different approaches described. Social role approaches could describe how the standards set by the medical profession (such as the need to pass formal exams, be recognized as competent by colleagues, etc.) can establish doctors as medical experts. Cognitive authority approaches will stipulate doctors’ superior epistemic status relative to novices. Capacity-based approaches will require doctors are actually able to help patients in some way (e.g. improve their health). Finally, performance-based approaches will look at the training doctors receive to determine whether or not it is appropriate for the development of expertise.

Regarding, epistemic authorities (a narrower and more demanding notion), a particular doctor would need to count as someone that a patient conscientiously takes to form beliefs (about medicine) via a mechanism that the patient trusts more than she trusts the mechanism by which she forms beliefs in that domain, to count as an epistemic authority.

Do doctors meet these requirements? At a first approximation, yes. Medical training, qualifications, and experience seem designed to ensure that doctors acquire the skills needed to interpret the clinical evidence base and form true beliefs in the domain of medicine. By virtue of this training and being accepted as members of the medical profession, credentialed healthcare professionals thus fulfil the performance-based and social role-based accounts of expertise. Since the clinical evidence base provides evidence of direct relevance to answering questions in medicine (i.e. counts as relevant evidence to patients making decisions), doctors’ training in interpreting and applying this evidence should lead them to form more true beliefs/make correct inferences and position them well to guide patients in their decision making. This points to the likelihood that doctors will satisfy cognitive authority and capacity-based approaches to expertise. Moreover, if doctors count as experts along these epistemological lines, then it seems likely patients will (and should) recognize them as epistemic authorities in the medical domain.

But the picture gets a little more complicated. In the following discussion, I don’t want to argue that medical doctors and their healthcare professional colleagues are *not* experts or epistemic authorities. Instead, I will motivate the concern that relying on their expertise – at least in some contexts – and deferring to their judgements is not as epistemically well-founded as one might assume and that this is troubling for autonomy.

The first thing I will consider is the fact/value distinction, and the extent to which doctors’ expertise covers the domain of interest (medical decision-making), given this domain involves answering hybridized questions that cover both ‘factual’ and ‘value’ content. Next, I will consider the extent to which doctors are well positioned to have good evidence regarding the ‘facts’ and are likely to form domain-relevant true beliefs. I then raise the issue of whether, even if doctors do have a good grasp of the facts and are likely to form true beliefs, they have the communication and other skills needed to support patients in using this information to make medical decisions. Finally, I consider how variability between different doctors’ skills and experience and propensity to form true beliefs and successfully communicate these to patients affects the capacity of patients to depend upon or defer to doctors as epistemic authorities in the context of medical decision-making.

Since my focus here is on medical decision-making, I am focusing more on those aspects of expertise commonly referred to as ‘cognitive’ expertise, rather than on the practical skills involved in being a doctor (e.g. performing surgery or conducting a physical examination).

### 3.1 The fact/value distinction as an obstacle to medical expertise

The fact/value distinction is long-standing, and pointed to as a reason why it is not possible to infer an ‘ought’ from an ‘is’. The fact/value distinction is generally applied in the realm of medical decision-making as justifying a need for healthcare professionals and patients to collaborate. Paternalistic decision-making, where doctors take a strong lead and present the ‘appropriate’ course of action, giving the patient little or no opportunity to consider alternatives, is generally rejected. First, most obviously, paternalism fails to respect patient autonomy. Further, it may fail to achieve good outcomes, as judged either by patients or doctors: without consulting patients on their values, doctors risk, for instance, recommending treatments that patients cannot adhere to, meaning they are unlikely to be successful. Or doctors may recommend treatments that fail to promote the things patients care about. The significance of patient values in determining success is neatly illustrated in cases of so-called ‘preference-sensitive’ decisions, where there is no clearly clinically preferable option, yet treatment options differ along dimensions that might influence patient preferences. Treatment for primary breast cancer is typically considered one such case: after diagnosis, women may receive a mastectomy (removal of the breast containing the tumour) or lumpectomy + radiation (where the surgical removal of tissue is less extensive, but must be followed up with additional radiation therapy to ensure all cancer cells have been destroyed) (Entwistle *et al*. 2014). Although neither treatment is considered clinically superior, women might have reasons (for instance aesthetic, or convenience) for preferring one or other treatment. Thus, doctors’ clinical expertise is insufficient to determine which treatment option is best.

Recognition that both patients and doctors bring relevant knowledge to decision-making processes has led to the development of models of ‘shared decision making’ where patients and their healthcare providers collaborate to understand the implications of different treatment options and settle on a course of treatment (or non-treatment) that best meets patients’ needs.^11^

One must be careful, however, of characterizing ‘collaborative’ decision processes like this as involving two parties who each bring distinct and limited forms of knowledge to the discussion. That is, it would be a mistake to assume that doctors’ knowledge is composed entirely of value-neutral facts and patients’ of fact-neutral values. As has been previously well-documented by sociologists and philosophers of scientific knowledge, the demarcation between facts and values is not a bright line, and neither is it the case that doctors have exclusive access to one and patients to the other.^12^

Facts can be ‘value-laden’ and (though it is less frequently discussed) values ‘fact-laden’ (Gorski 2013). Although this recognition is almost as commonplace as the initial fact/value distinction, it is worth emphasizing just how deep it goes. Scientific research and clinical guidelines rely heavily on a range of judgements. All sorts of decisions must be made when designing, executing, and interpreting clinical trials, including the appropriate participant population, details of the intervention (and control arm, if present), suitable outcome measures, how to design the statistical analyses, writing up, and sharing of results. Decisions, such as the norm within some scientific disciplines to adopt a p value of 0.05 as indicative of ‘statistical significance’, are not purely scientific decisions, but are decisions made while reflecting upon, for instance, the appropriate degree of sensitivity we should have to different kinds of errors – whether it is better to falsely reject or falsely accept a null hypothesis. The risk of such errors is known as ‘inductive risk’. Scientific methodology is unavoidably infused with these kinds of judgements. Douglas (2000) argues that the presence of inductive risk means that, whenever non-epistemic consequences are at stake as a result of scientific error, non-epistemic values must be considered as a part of the internal process of scientific research itself. For instance, when considering whether to offer a screening intervention, one should consider the harms of unnecessary treatment that could result from a false positive test result, along with the harms of a false negative (i.e. failing to spot pathology that is present).

To describe these judgements as ‘value-laden’ is not, typically, to claim that they are justified by some ethical theory or informed by explicit moral reasoning. Often the values at issue appear scientific in nature: how to best design a scale for measuring changes in depression or cognitive function; how the control arm of a trial can best be crafted to isolate the effects of interest of the intervention; what sample size to use. Yet these are, in a broad sense, value judgements since they depend upon the subjective assessment of the researcher and will be informed by her goals. That is not to say that all such judgements primarily result from the deliberations of each individual researcher: many such decisions are guided by scientific norms of practice (such as the aforementioned threshold of p = 0.05 for statistical significance). Nor that they are necessarily particularly controversial (such as the practice of excluding pregnant women or those with pre-existing conditions from phase 1 trials).

Why think that this a problem? Such decisions must be taken, and scientific expertise can help to inform them. Moreover, scientists involved in the design, execution, and analysis of trials can take steps to inform such decisions (through practices such as patient and public involvement). The problem arises when the influence of these kinds of judgements is widely under recognized (or considered ‘scientific’ in nature) and where they filter through to influence patient decision-making in ways that render patients’ decisions significantly influenced by others’ values, rather than their own. One particular cause for concern is if the goals (due to financial and other incentives, including particular value commitments) of researchers, healthcare professionals, and health policy makers systematically differ from those of patients. It would be surprising if, for instance, those working in public health promotion didn’t tend to value public health at least a bit more than the average member of the public (who might place relatively more weight on the value of pleasures like alcohol consumption and tasty food). Doctors’ risk tolerance might also differ from that of their patients, meaning their recommendations are mis-tailored to their patients’ preferences in ways that are not apparent to either party.

Researchers, clinicians, and policymakers also have their own, perhaps less laudable, incentives – to publish impressive papers, to deal with patients as quickly as possible, to introduce vote-winning policies. In the clinical research context, financial conflicts of interest have been fairly well documented, and steps taken in efforts to neutralize them, but it seems they are likely to still wield a pernicious influence (Howick 2019). Pressure to find ‘positive’ and impressive results can also lead scientists to exaggerate the significance of their data and engage in bad practice, up to and including creating fraudulent data (Ritchie 2020). The result is an evidence base that systematically over-estimates the benefits (and underestimates the harms) of medical interventions (Brown *et al*. 2022; Stegenga 2018).

When patients must rely upon doctors’ testimony for making medical decisions, they are placed in what Guerrero (2016) calls ‘strategic expertise contexts’. These are contexts where there is asymmetric expertise and thus asymmetric ignorance between two parties, and where ‘there is some measure of non-alignment’ (p. 157) between the expert’s and non-expert’s interests. This provides some reason for the non-expert to not completely trust the expert. As outlined by Guerrero, many of the strategies novices have to evaluate experts as to their competence and integrity (including gaining insight into the values and motivations of experts) are severely limited (Guerrero 2016).

Much medical practice is ‘effective’ according to fairly uncontroversial standards (i.e. it clearly improves things that people care about such as pain, duration of infection, life expectancy, etc.). Despite the flaws mentioned, evidence-based medicine has been hugely important in developing effective treatments. Yet for areas of medicine where the gains of treatment relative to harms are more marginal (and where the cost of treatment is high), the biases arising in clinical research and practice can disguise the fact that interventions are all-things-considered ineffective or harmful. Moreover, over-optimism about the effectiveness of interventions is likely to lead to unrealistic expectations and poorly informed decision-making (Hoffmann and Del Mar 2015, 2017).

### 3.2 How impressive are doctors’ skills and evidence?

Even if we set aside the concern that many clinical judgements involve (proximal and distal) value-judgements, it may be the case that doctors’ grasp of what are taken to be medical facts is not as strong as one might expect. Although the superiority of their skills in interpreting clinical evidence and access to appropriate evidence may be taken for granted, it is worth considering just how superior those skills and evidence are.

First, it is worth acknowledging that evidence-based medicine is undoubtedly a vast improvement on the techniques of observation and guesswork that preceded it. The development and scrutiny of clinical trial methodologies has enabled a far better understanding of the underlying causes of a range of health conditions and the harms and benefits of techniques used to treat them. A recent vivid example is the successful development of a (number of) safe and effective vaccine(s) to protect people from the SARS-CoV-2 virus. Without losing sight of the gains enabled by evidence-based medicine, we should recognize continued weaknesses in the processes of researching and implementing effective healthcare interventions.^13^

As mentioned in the previous subsection, current practices of clinical research lead to systematic overoptimism regarding the harms and benefits of medical interventions. The factors contributing to this have been convincingly articulated by Stegenga (2018) who advocates for a stance of ‘medical nihilism’.^14^ Others have also discussed, in less provocative language, reservations about the continued flaws in evidence-based medicine (Howick 2019; Mandrola *et al*. 2019; McCartney 2012). Some of the concerns relate to statistically significant but clinically meaningless benefits being identified; a vast volume of evidence being produced; poor methodologies (such as biased trial designs and the adoption of inappropriate measures); the unknown extent of fraudulent research findings; poor adherence to pre-registration requirements/non-publication of ‘negative’ results, and so on (see also (Brown *et al*. 2022)). The problems with the quality, applicability, and usability of the clinical evidence based cannot always be simply resolved by recourse to selective aggregative methods such as systematic reviews and meta-analyses. As the adage goes: garbage in, garbage out.

Clinical evidence will inform the beliefs (and practice) of doctors and other healthcare professionals in more and less direct ways. Doctors can observe the effects of treatments they provide to their patients and extrapolate from this to form expectations about the effects of treatments on other patients; they may be involved in conducting formal clinical research; they will read or hear about clinical research or colleagues’ experiences with particular treatments; they will receive training on the appropriate course(s) of treatment for particular conditions; they will be provided with guidelines as to the treatment recommendations (and restrictions on what is available) in given circumstances.

Although there are problems with the quality of the clinical evidence base and the applicability of research findings to patient populations, published clinical research nonetheless often represents the most reliable evidence available regarding the likely effects of particular treatments. Yet doctors may lack the necessary skills to interpret such evidence correctly. A body of research has established that doctors receive inadequate training in medical statistics and risk communication, and often lack basic numeracy (Altman and Bland 1991; Gigerenzer and Gray 2013). For instance, when asked three simple questions (including converting a percentage to a number out of 1,000 and doing the same in reverse) between 28% and 40% of doctors answered at least one question incorrectly (Estrada *et al*. 1999; Wegwarth and Gigerenzer 2011). One area where doctors may well be called upon to advise patients is regarding screening and diagnostic testing, which requires the capacity to interpret and communicate concepts such as baseline risk, test sensitivity, specificity, and positive predictive value. Unfortunately, it seems many doctors lack the skills needed to interpret screening test data appropriately. That is, they are unable to correctly assess the likelihood that someone who receives a positive test *actually has*, e.g. cancer and vice versa. This has been shown in a number of different screening contexts, including Down’s syndrome, mammography, bowel cancer screening, and HIV screening (Bramwell *et al*. 2006; Eddy 1982; Gigerenzer *et al*. 1998; Hoffrage and Gigerenzer 1998). Not only may doctors struggle to correctly interpret test results but the embarrassment they feel at their self-perceived ‘innumeracy’ may lead them to avoid revealing their lack of understanding to patients by avoiding mentioning any numbers during consultations (Hoffrage and Gigerenzer 1998; Wegwarth and Gigerenzer 2011).

The difficulties doctors have with interpreting and communicating risk information may stem from a lack of training in medical statistics (and lack of access to competent statistical advisers) (Altman and Bland 1991), as well as a polluted epistemic environment, with limited time and resources to overcome this. Risk information is often presented in journal articles and informational leaflets in ways that will predictably mislead. For instance, relative risks are often used to describe the benefits of interventions, rather than the more transparent absolute risks; benefits reported in relative risks are often paired with harms reported in absolute risks (exaggerating the misinterpretation of these statistics) (Sedrakyan and Shih 2007). Similar patterns are found in public health information published online (de Barra and Brown 2023). There have also been various problems identified with the contents of randomized controlled trials and systematic review abstracts, which clinicians often rely upon for making clinical decisions about interventions (Nascimento *et al*. 2021).

I have focused here on those skills and evidence that relate to doctors’ cognitive authority, specifically their ability to interpret the clinical evidence base and their facility with statistical information. There are many other skills that doctors and other healthcare professionals possess – performing routine procedures, diagnosing common conditions, correctly prescribing well-used medications – which may be reliably performed. By discussing areas of weakness I do not intend to give an overly pessimistic impression. It is, however, important to attend to areas where doctors fall short of expectations, and where their capacity to usefully guide medical decision-making is limited, when considering the extent to which they are experts to whom patients can comfortably defer.

### 3.3 How good are doctors at communicating relevant information?

Doctors not only need skills to correctly interpret medical evidence but they must also be able to effectively communicate the significance of such evidence to patients in the context of medical decision making. Mentioned above was the lack of statistical training and support provided to doctors. It seems that the same is true for risk communication (Gigerenzer *et al*. 2007).

Effective clinical communication skills support effective diagnosis; patient satisfaction and understanding; adherence to treatment plans; reduce patient distress, anxiety, and depression; and improve doctors’ own well-being (Maguire and Pitceathly 2002; Maguire *et al*. 1986; Parle *et al*. 1996; Ramirez *et al*. 1996; Roter *et al*. 1995; Silverman *et al*. 2016). As described by Maguire and Pitceathly (2002), doctors’ communication skills are often deficient:

Only half of the complaints and concerns of patients are likely to be elicited… Often doctors obtain little information about patients’ perceptions of their problems or about the physical, emotional, and social impact of the problems… When doctors provide information they do so in an inflexible way and tend to ignore what individual patients wish to know. They pay little attention to checking how well patients have understood what they have been told… Less than half of psychological morbidity in patients is recognised. (p. 697)

Doctors (and patients) also often leave out information relevant to decision making, which may well influence patient decisions (Bugge *et al*. 2006). One risk of doctors’ failure to communicate relevant information is the removal of important choices. In the worst cases, this can result in severe health harms, as in the case of *Montgomery vs Lanarkshire 2015*. This was an obstetric case, where the doctor (Dr McLellan) failed to disclose the risk of shoulder dystocia to a woman (Mrs Montgomery) due to give birth. Dr McLellan was explicit that her reason for not disclosing this risk was that it was likely to make Mrs Montgomery prefer a caesarean section, which the doctor did not consider was in the patient’s interest. The attempted vaginal delivery did result in shoulder dystocia which led to hypoxic injury and cerebral palsy in the baby. The Montgomery case led to a reform of British law as regards requirements for disclosure in medical decision making.

At the other end of the spectrum, poor judgement regarding informational needs (perhaps combined with a fear of falling foul of disclosure requirements) can lead to ‘information dumping’. This overwhelms patients with medical details that they cannot interpret and makes it hard to distinguish the relevant from the irrelevant.

### 3.4 Variability in doctors’ skills and evidence

It is perhaps worth noting – briefly – the obvious: that doctors’ (and other healthcare professionals’) skills and evidence will vary across individuals. Although reassurance can be gleaned from the standardized training and assessment of healthcare professionals, this will still leave significant room for variation between individuals, both in terms of their skills in interpreting and communicating evidence and their capacity to access evidence. There will also be variation within individuals (across their careers, as well as, presumably, over a shorter timescale – a bad nights’ sleep must surely disrupt a doctor’s cognitive capacities much as it does a philosopher’s). Other factors may influence the kind of advice and information a doctor provides in clinical consultations, such as religious faith and ethnicity (Seale 2010).

It may be possible to judge whether the doctor we are currently dealing with has the relevant skills and evidence available to her or to surmise how personal characteristics or situational factors are influencing her judgement or communication regarding treatment options. Yet this may only be possible when very obvious (say, if a doctor seems excessively harried, appears confused during a consultation, or makes claims that are obviously incorrect or inconsistent), or when patients have a peculiar degree of insight into such things. At the more subtle level of judging expertise, those who lack knowledge in a particular domain will struggle to judge others’ knowledge accurately (Ballantyne 2022; Dunning and Cone 2018; Guerrero 2016).

## 4 Circumscribed expertise

It would be extravagant to conclude based on the above discussion that doctors and other healthcare professionals do not count as experts, or that they (should) fail to be recognized as epistemic authorities for particular patients. Instead, I think it shows that their expertise is likely to be less robust than is commonly assumed. What does this mean? Recall, expertise may be defined in a variety of ways. Promising approaches relevant to the kind of expertise of interest here – that is, expertise that can be used to guide medical decision-making – include possessing cognitive authority as a result of having more true beliefs (and fewer false ones) than most people; or having access to more reliable evidence; or displaying an understanding of and ability to work with the concepts and practices within a domain (Goldman 2018; Watson 2020). This includes possessing the necessary skills to interpret the relevant evidence (Ballantyne 2019). Alternatively, experts might be judged according to their capacity to help others solve problems or execute tasks (Goldman 2018) or might need to have undergone appropriate training.

This discussion suggests that doctors’ cognitive authority is weaker than typically assumed. Limitations in training and pollutants in the clinical evidence base mean doctors will have fewer true beliefs (and more false beliefs) than we ordinarily assume, and that they hold these with an inappropriate degree of confidence. The same goes for their possession of more and better evidence than most people: whilst doctors are ordinarily better able than patients to interpret clinical evidence and have access to medical guidelines, this evidence and the guidelines based upon it are flawed, and doctors’ ability to interpret clinical evidence and other statistical information is also limited. This limited expertise might also be reflected in doctors’ training: as discussed, training in statistical inference and interpretation of clinical evidence is minimal for medical students.

I have so far avoided too much discussion of the domain within which doctors and other healthcare professionals are to be considered experts. If we take the domain to be ‘medicine’ broadly, it is easier to make the case for their expertise. But when considering patient autonomy with regard to medical decision-making, we may want to be narrower. This is particularly the case if we consider the question of whether or not a given doctor can act as an epistemic authority for a particular patient with regard to a particular medical decision. Matters will vary from decision to decision: some medical decisions are relatively straightforward and have good evidence to inform them, of which doctors are well aware; others lack high-quality evidence and, more troublingly, there is a lack of awareness of the poor quality of that evidence. Once we weaken the extent to which doctors are assumed to have the relevant skills, true beliefs, or understanding regarding a particular medical decision, we also weaken the case for preemptive adoption of doctors’ beliefs. The domain within which healthcare professionals are asked to act as experts (particularly where they are generalists and must cover a very wide range of medical questions) includes matters on which they *lack* expertise (based on my discussion of the depth of the blurring between facts and values in ‘scientific’ research). Skilled practitioners here will recognize when they encounter issues outside their expertise but, as discussed by Dunning (2022) it can be difficult to know what you don’t know.

And the foregoing discussion might even present too rosy a picture of healthcare professionals’ capacities to exercise relevant expertise. It has lacked any consideration of the particular strains that doctors and other healthcare workers are often placed under, and which makes it difficult for them to manifest the skills and attend to the evidence needed to inform patient decisions. Doctors are often time-pressed (general practitioners in the UK and elsewhere typically have 10–15 mins per appointment, and in other contexts have to ration their time between different patients) and the nature of healthcare work can lead to stress and burnout among staff and result in poorer quality care (Firth-Cozens 2003; Jones *et al*. 1988; Mollart *et al*. 2013). Such factors could limit healthcare professionals’ capacity to support medical decision-making effectively.

## 5 Autonomous deference

We began with the question of whether or not patients could maintain their autonomy while being dependent on their doctor or other healthcare provider for relevant knowledge. The answer to this was ‘yes’ since (a) good epistemic practice is a part of autonomous action, and deferring to experts is good epistemic practice; (b) autonomy doesn’t require acting completely independently; and (c) the testimony of experts can be treated like other forms of evidence, and factored into an individual’s decision-making process as she would factor in other evidence.

But having considered a number of ways in which doctors’ expertise seems lacking, we now arrive at a slightly different question: can patients maintain their autonomy whilst being dependent on healthcare providers *given* that the expertise of such professionals is not as robust as people might expect? There are two forms the threat to autonomy could take here: (1) depending on doctors whilst having an inflated expectation of their expertise, and (2) depending on doctors even once one has a more realistic understanding of their expertise.

The first form seems reasonably straightforward, insofar as it involves a false belief, which is *prima facie* concerning for autonomy. I will assume that autonomy is graded, in which case having more false beliefs, particularly about matters centrally important to the decision at hand, will tend to reduce autonomy. Beliefs about the expertise of one’s healthcare provider seem centrally important to medical decision making. If one has inflated expectations of doctors’ skills and evidence, one will treat their testimony as more likely to be epistemically well-grounded, and more likely to track the truth, than it in fact is. One may also judge doctors to be epistemic authorities – and potentially adopt their beliefs pre-emptively – in situations where this is inappropriate.

Trust in scientists and healthcare professionals is generally high globally (Ipsos 2019). British respondents put nurses top when asked if they would generally trust [profession X] to tell the truth (Ipsos 2022); doctors were third and scientists fourth. Perceived competence, along with benevolence, are used by people to judge whether or not to trust a particular source (Mercier 2020), suggesting that healthcare professionals are generally judged to be competent. Believing someone to be more expert than they in fact are – believing their skills and evidence to be superior – threatens autonomy, since one will over weight their testimony as evidence.

The second form is less straightforward. Here, let us assume that patients have an accurate picture of the limits of doctors’ and other healthcare professionals’ expertise. They are aware of the weaknesses I have pointed out above, correctly judge the extent to which doctors training and experience equip them with skills and evidence that are superior to the patient’s own, and the extent to which doctors will have more true (and fewer false) beliefs than the patient themselves has. They recognize the extent to which doctors’ belief forming processes in the relevant domain are more reliable than their own belief forming processes. But what should the patient do now? It seems that even recognizing the degree to which controversial value judgements are incorporated into medical evidence and recommendations, deficiencies in the clinical evidence base, doctors’ capacity to interpret, apply, and communicate this evidence, and the variability across different doctors, patients are still in a position where they are better off deferring to medical opinion.

Now, however, they are deferring to an opinion that they know is less likely to be ‘right’ – it is less likely to reflect the way the world is, to improve their health or track their values. Since these patients are better informed about the quality of the (testimonial) evidence upon which they are relying, they will be more autonomous than those who remain ignorant. Such enlightened patients may even feel doctors’ expertise does not warrant deferral (that neither preemption nor a more moderate form of deferral is appropriate). If they wish to act contrary to medical advice, however, they may face barriers. Whilst patients have the legal right to refuse consent to interventions, they may often still be in a relatively weak position with regard to accessing preferred alternatives or receiving suitable diagnoses (see, for instance, debate around provision of caesarean sections without ‘medical indication’, or deep dissatisfactions amongst people suffering with myalgic encephalomyelitis/chronic fatigue syndrome (Byrne 2020; Romanis 2019)). Heavy legal weight is given to clinical judgement and although a given patient may believe that her medical team is offering inappropriate advice, the social and institutional pressure to nonetheless comply with their recommendations can be powerful.

## 6 Concluding remarks

The mere fact that patients must rely upon medical expertise when making medical decisions does not unduly threaten their autonomy. Yet concerns about autonomy arise when we consider how robust that expertise is, the extent to which patients are misinformed about the quality of healthcare professionals’ expertise, and the (genuine) opportunities patients have to make decisions which go against medical advice. I suspect that evaluations of doctors’ expertise are typically inflated (both by patients and by doctors themselves), due to a lack of awareness of the limitations on the quality of the clinical evidence base and doctors’ skills at interpreting this evidence and communicating it to patients. I also suggest that it is difficult for patients to do other than defer to medical advice, because (1) *even though* the medical expertise they encounter may be significantly flawed, it is likely to still be superior to their own, and (2) because it is in practice hard to act contrary to medical advice, requiring an unusual degree of assertiveness and self-confidence. This creates problems for patient autonomy.

Solutions that might help to bolster autonomy include those directed at improving medical expertise directly: efforts to improve the methods and quality of the clinical evidence base, and healthcare professionals’ skills at interpreting and communicating this evidence. There is also precedent for bolstering patient expertise: in the UK, the ‘Expert Patients Programme’ provides training for patients with long-term conditions to teach them how to better manage their conditions and monitor their symptoms (UK Government 2013). Patient expertise is an under analysed concept, but recent work has sought to better conceptualize and integrate it alongside medical expertise (Watson 2024). There are also established ‘best practices’ for facilitating patient involvement in their care and shared decision making, including communication techniques to promote health literacy (by, for example, avoiding medical ‘jargon’, using ‘teach back’ techniques to correct misunderstandings, etc.) and the use of decision aids (Coleman *et al*. 2017). Such tools and techniques can supplement medical expertise, help patients to appropriately incorporate evidence into their decision making and enable them to make value-congruent decisions.

In addition, promoting epistemic humility and helping both healthcare professionals and patients to calibrate their confidence in medical expertise will improve everyone’s epistemic standing and reduce the tendency to defer too readily to medical expertise. Such approaches treat experts as advisors rather than authorities, as Lackey (2018) recommends, and reduce inappropriate deference. Patients might also seek second opinions to introduce more diversity of evidence and robustness into the decision process. These strategies will work best when they are not seen as undermining doctors’ professional expertise but are understood to reflect the inevitable limitations of expertise in contexts where information is complex and uncertain.

Finally, a more realistic recognition of the limitations of medical expertise should be reflected in the operation of legal, social, and medical institutions, such that clinical judgement is not given an inappropriate degree of weight (and patient judgement is not similarly misjudged).

Patients are unavoidably dependent upon medical experts when making decisions about their health. Such expertise is inevitably limited and this can threaten patient autonomy, particularly if they are unaware of the limitations of the expertise on which they rely, or if they are aware of those limitations but disempowered to make decisions contrary to medical advice. However, integrating medical expertise into decision making can be autonomy-promoting when patients and doctors are aware of the limitations and extent of their own and each others’ expertise, and this is reflected in the way expertise informs the final decision.^15^

## Footnotes

1 This isn’t necessarily the case. Patients can be very well-informed regarding their condition – perhaps moreso than their clinical team – and can contribute to clinical research and practice in important ways (see Epstein 1996; Watson 2024).

2 Perhaps it is more balanced to suggest such contexts present opportunities to promote autonomy as well. To the extent, however, that we are reasonably more concerned about losses of autonomy than opportunity costs, it may be fair to focus on the threats rather than opportunities posed by medical decision-making.

3 Levy (2022) similarly thinks that ‘doing your own research’ rather than simply deferring to experts can enhance understanding, and thus be of value. Levy is, however, unconvinced that routinely relying upon one’s own belief-forming methods (rather than exclusively relying upon experts) will tend to promote true beliefs.

4 Thanks to an anonymous reviewer for helpful clarification on this point.

5 Thanks to a reviewer for highlighting this aspect of relational autonomy.

6 More strictly it involves a belief or an ‘optimistic attitude’. An optimistic attitude that P falls just short of a belief that P, but involves not “entertaining as a live epistemic possibility that not-P.” (Fricker 2021*a*, 2021*b*: 61–62)

7 For further discussion see Sperber *et al*. (2010) and Mercier (2020) on ‘epistemic vigilance’ and trust.

8 Fricker’s account might also undermine the threat preemption poses to autonomy directly, by suggesting preemption is not even epistemically required: Fricker’s view recommends treating expert testimony as strong evidence, which should be integrated with our existing reasons, rather than treating it as preempting our reasons.

9 This is a slight deviation from Zagzebski’s preferred definition. Zagzebski emphasises that epistemic authority is concerned with forming beliefs *conscientiously*. Thus, she cashes out the justification for the authority of another’s belief as arising from “my conscientious judgment that I am more likely to form a belief that survives my conscientious self-reflection if I believe what the authority believes than if I try to figure out what to believe myself.” (Zagzebski 2012: 110–11)

10 For domains where expertise is largely cognitive (including the domain at issue – medical decision-making), what Watson describes as ‘epistemic facility’ (i.e. the cognitive authority component of his account) will largely explain what competence involves.

11 There has been much discussion of shared decision-making in medicine, see: Barry and Edgman-Levitan (2012), Epstein and Gramling (2012), Munthe *et al*. (2012), Elwyn *et al*. (2016).

12 See also Watson’s (2024) discussion of patient expertise.

13 In these sections I discuss both clinical research and practice. Although clinical research is not directly translated into practice, it influences it in both direct and indirect ways (as discussed below). It is healthcare professionals’ access to and ability to interpret and apply clinical evidence that forms a core basis of their cognitive expertise and authority. Although I recognize that some of the flaws introduced during clinical research can be corrected for at the clinical level, guidelines and best practice are heavily informed by the clinical evidence base and, to the extent that the latter is distorted, the former will be polluted.

14 It is worth noting that Stegenga’s critiques focus mostly on recent pharmaceutical developments which appear to offer small if any gains over existing therapies.

15 This research was funded in whole or in part by UKRI (AHRC), Grant number AH/W005077/1 and the Wellcome Trust, Grant number WT203132/Z/16/Z.). For the purpose of Open Access, the author has applied a CC BY public copyright licence to any Author Accepted Manuscript (AAM) version arising from this submission. Thanks to Neil Levy for helpful comments on the manuscript.
